# Forest Walking Affects Autonomic Nervous Activity: A Population-Based Study

**DOI:** 10.3389/fpubh.2018.00278

**Published:** 2018-10-01

**Authors:** Hiromitsu Kobayashi, Chorong Song, Harumi Ikei, Bum-Jin Park, Juyoung Lee, Takahide Kagawa, Yoshifumi Miyazaki

**Affiliations:** ^1^Department of Nursing, Ishikawa Prefectural Nursing University, Ishikawa, Japan; ^2^Center for Environment, Health and Field Sciences, Chiba University, Kashiwa, Japan; ^3^Forestry and Forest Products Research Institute, Tsukuba, Japan; ^4^Department of Environment and Forest Resources, Chungnam National University, Daejeon, South Korea; ^5^Department of Landscape Architecture, Hankyong National University, Anseong-si, South Korea

**Keywords:** forest therapy, walking, heart rate variability (HRV), skewness, kurtosis, population approach

## Abstract

The present study aimed to evaluate the effect of walking in forest environments on autonomic nervous activity with special reference to its distribution characteristics. Heart rate variability (HRV) of 485 male participants while walking for ~15 min in a forest and an urban area was analyzed. The experimental sites were 57 forests and 57 urban areas across Japan. Parasympathetic and sympathetic indicators [lnHF and ln(LF/HF), respectively] of HRV were calculated based on ~15-min heart rate recordings. Skewness and kurtosis of the distributions of lnHF and ln(LF/HF) were almost the same between the two environments, although the means and medians of the indicators differed significantly. Percentages of positive responders [presenting an increase in lnHF or a decrease in ln(LF/HF) in forest environments] were 65.2 and 67.0%, respectively. The percentage of lnHF was significantly smaller than our previous results on HRV during the viewing of urban or forest landscapes, whereas the percentage of ln(LF/HF) was not significantly different. The results suggest that walking in a forest environment has a different effect on autonomic nervous activity than viewing a forest landscape.

## Introduction

“Shinrin-yoku” is a Japanese term for “forest bathing,” which was coined by the Director of the Japanese Forestry Agency, Tomohide Akiyama, in 1982 (1). This term is now increasingly being used internationally (1–3). Various studies on the psychological effects of natural environments have been conducted, with consistent effects of reducing negative emotions, such as anger, fatigue, or sadness, being demonstrated in previous studies (4). In addition to psychological effects, beneficial effects of a forest environment in terms of physiological responses have also been investigated (5). Decreases in blood pressure (6–8), in serum or salivary cortisol concentration (6, 9, 10), and enhancements in immune system functioning (11–13) have been reported.

Heart rate variability (HRV) measurement is a method for evaluating autonomic nervous functions. HRV measurement is the most frequently used physiological indicator in studies on the effect of forest environments and demonstrates better results than other physiological measurements, such as salivary cortisol concentration (10). The power spectrum of the heartbeat interval sequence generally exhibits two spectral components: a high-frequency (HF; 0.15–0.40 Hz) component and a low-frequency (LF; 0.04–0.15 Hz) component. The HF component of HRV is considered to be a marker of parasympathetic activity, whereas the LF component or LF/HF ratio is considered to be a marker of sympathetic activity (14, 15). Several studies have consistently demonstrated increases in HF and/or decreases in LF/HF in forest environments compared with the corresponding levels in urban environments (16–18). These results suggest that being present in a forest environment relaxes the autonomic nervous system.

HRV measurements have the advantages of enabling continuous ambulatory monitoring and robustness against artifacts, such as body movement. These advantages might be maximized in measurement performed during walking in a field environment rather than during resting in laboratory condition. HRV measurements have also been applied in studies on the effects of walking in natural environments (19–21), which also reported relaxation of autonomic nervous system in forest environments similar to that in studies conducted on a resting condition. The present study investigated the HRV of 485 young male participants during walking in forest and urban environments.

In efforts to promote human health, there are two types of strategy: a high-risk (individual) approach and a population approach. The high-risk approach targets individuals with a certain disease or impairment, whereas the population approach targets an entire population. Nature therapy, including “shinrin-yoku,” is one of the population approaches to promote health. Although its effects on each individual are relatively small, at the population level, it can achieve greater health improvement by shifting the risk distribution curve of the entire population (22). Thus, the beneficial effect of exposure to the natural environment should be evaluated using a population-based analysis with special reference to its distribution characteristics. However, most previous studies on nature therapy have merely focused on the change in the mean values of health-related variables [e.g., (6–13)]. To adopt a population-level perspective, in this study, we analyzed HRV indicators of 485 male participants with special reference to their distribution characteristics. In addition, we compared our results obtained during walking with those during the viewing landscapes reported in a previous study (17).

## Materials and methods

### Study sites and participants

The study areas were 57 forests and 57 urban sites across Japan. Urban areas were downtown or near a Japan railway station. Although 684 young (aged 19–29 years) Japanese male university students participated in the experiments, only 520 participants whose complete data could be obtained at both forests and urban sites were analyzed. Demographic parameters of the participants are shown in Table 1. None of the participants reported a history of physical or psychiatric disorders. During the study period, alcohol and tobacco consumption was prohibited and caffeine consumption was controlled.

**Table 1:** Demographics of the participants (*n* = 520).

	**Age (year)**	**Height (m)**	**Body mass (kg)**
Max	29	1.88	110
Min	19	1.55	42
Mean	21.7	1.72	64.6
SD	1.6	0.06	9.5

*SD, standard deviation*.

### Experimental design

The experiment was performed at each experimental area over 2 consecutive days. Prior to the experiment, the aim of this study and the experimental protocol was explained and general instructions were provided to the participants. The participants participating in an experiment at each site were randomly divided into two groups, and the order of the experimental conditions (urban or forest) was counterbalanced among them. One group performed the experiment in the forest area prior to the urban area, and the other group performed the same experiment in the urban area prior to the forest area. All participants stayed in a waiting room before moving to the field site. All participants were instructed to rest in a chair for ~5 min, which mitigated the physiological effects of physical activity before the measurement period. The HRV data were obtained during walking in a forest or an urban environment for ~15 min. On the second day, the participants switched field sites. The experimental protocol for the second day was the same as the first day.

Among the experiments at 57 locations, those at 44 locations were performed with the experimental design of “Stay-in Forest Therapy,” in which all participants were instructed to reside in a hotel with identical single rooms. Meanwhile, the experiments at 13 locations were performed with the experimental design of “One-Day Forest Therapy,” in which the participants returned home after the first day of the experiment. To reduce the burden on participants and the research expenses, eventually all experiments were switched to the simplified experimental design of One-Day Forest Therapy.

### HRV measurements

HRV was measured using a portable electrocardiograph (Activtracer AC-301A; GMS, Japan). Spectral analyses of HRV in 15-min recordings were conducted using HRV software (MemCalc/Win; GMS, Tokyo, Japan) based on the maximum entropy method. HF and LF components were obtained by integrating the power spectra at their respective ranges of 0.15–0.40 and 0.04–0.15 Hz. The natural logarithms of the HRV indices [lnHF, ln(LF/HF)] were then calculated because it has been reported that the raw HRV components exhibit skewed distributions (23).

In this study, HRV was measured during spontaneous breathing, and paced breathing was not applied. The participants were instructed to avoid irregular breathing during the measurements. A previous study reported that the effect of paced breathing on inter-individual variations in the spectral components of HRV was negligible (24).

### Outlier processing

Outlier processing was performed on the results because higher-moment statistics (skewness and kurtosis) are particularly sensitive to outliers (25). The outlier processing was based on a box-whisker plot (26). Upper and lower cut-offs (upperCO and lowerCO, respectively) were defined as follows:

UpperCO = Q3 + 1.5 (IQR), (1)
LowerCO = Q1 – 1.5 (IQR), (2)

where

Q1: quartile 1 (25th percentile)
Q3: quartile 3 (75th percentile)
IQR: interquartile range (Q3–Q1)

The outlier processing was performed on the HRV indices [lnHF and ln(LF/HF)] obtained in each environment (urban and forest). The lowerCOs and upperCOs are summarized in Table 2.

**Table 2:** Cut-off values of heart rate variability for the outlier processing.

	**lnHF**		**ln(LF/HF)**	
	**Urban**	**Forest**	**Urban**	**Forest**
LowerCO	0.85	1.09	0.49	0.22
UpperCO	6.95	7.54	3.83	3.76

*CO, Cut-off value for the outlier processing*.

The participants associated with outliers in either environment were eliminated. As a result, 35 participants were eliminated, and the data of the remaining 485 participants were used for further analysis.

### Statistical analysis

HRV indicators of the 485 participants were plotted as histograms by dividing the range [from 1.0 to 7.5 for lnHF, from 0.0 to 4.0 for ln(LF/HF)] into 40 segments. Changes in HRV indices between urban and forest environments (forest–urban) were also plotted as a histogram by dividing the range [from −4.5 to +5.5 for lnHF, from −3.0 to +2.0 for ln(LF/HF)] into 40 segments.

The mean, median, standard deviation (SD), coefficient of variation (CV), IQR, skewness, and kurtosis of the distribution were calculated. Skewness is a measure of the symmetry of distribution. Negative or positive skewness is indicated when the left or right tail, respectively, of the research data in a histogram is longer than the other tail. The skewness of a normal distribution is zero. Meanwhile, kurtosis is a measure of whether the distribution curve is peaked (positive) or flat (negative) relative to the normal distribution. The kurtosis of normally distributed data is defined as zero.

Differences in these statistics between urban and forest environments were tested by performing a permutation test, which is a statistical test with a non-parametric basis. Resampling was performed 5,000 times. The *p*-value was calculated according to the suggestion by Phipson and Smyth (27). The uncertainty of a *p*-value near 0.05 was estimated to be 0.3%.

For further analysis, results of this study were compared with those of our previous study (17). In the previous study, autonomic responses to urban and forest environments were studied in 625 young male participants. The participants viewed the landscape (forest or urban environment) for 15 min while sitting on a chair. When viewing the landscapes, HRV was monitored continuously.

Number of participants who indicated positive or negative responses were calculated for present (walking) and the previous (viewing) results. Positive and negative responses to forest environments were defined as a decrease in lnHF and an increase in ln(LF/HF), respectively. The difference between the present and previous studies with respect to the ratio of negative/positive responders was compared using Chi-squared test. *p*-values < 0.05 were considered indicative of statistical significance for permutation and Chi-squared tests.

## Ethical considerations

The study was conducted in accordance with the Declaration of Helsinki, and the protocol was approved by the Ethics Committee of the Forestry and Forest Products Research Institute, Japan (project identification code number: 16-558), or the Center for Environment, Health and Field Sciences, Chiba University, Japan (project identification code number: 5). Participants were informed about the purposes and procedures of the study and provided written informed consent prior to enrollment. They were free to not attend or cease participation in the program at any time.

## Results

**Figure 1:**
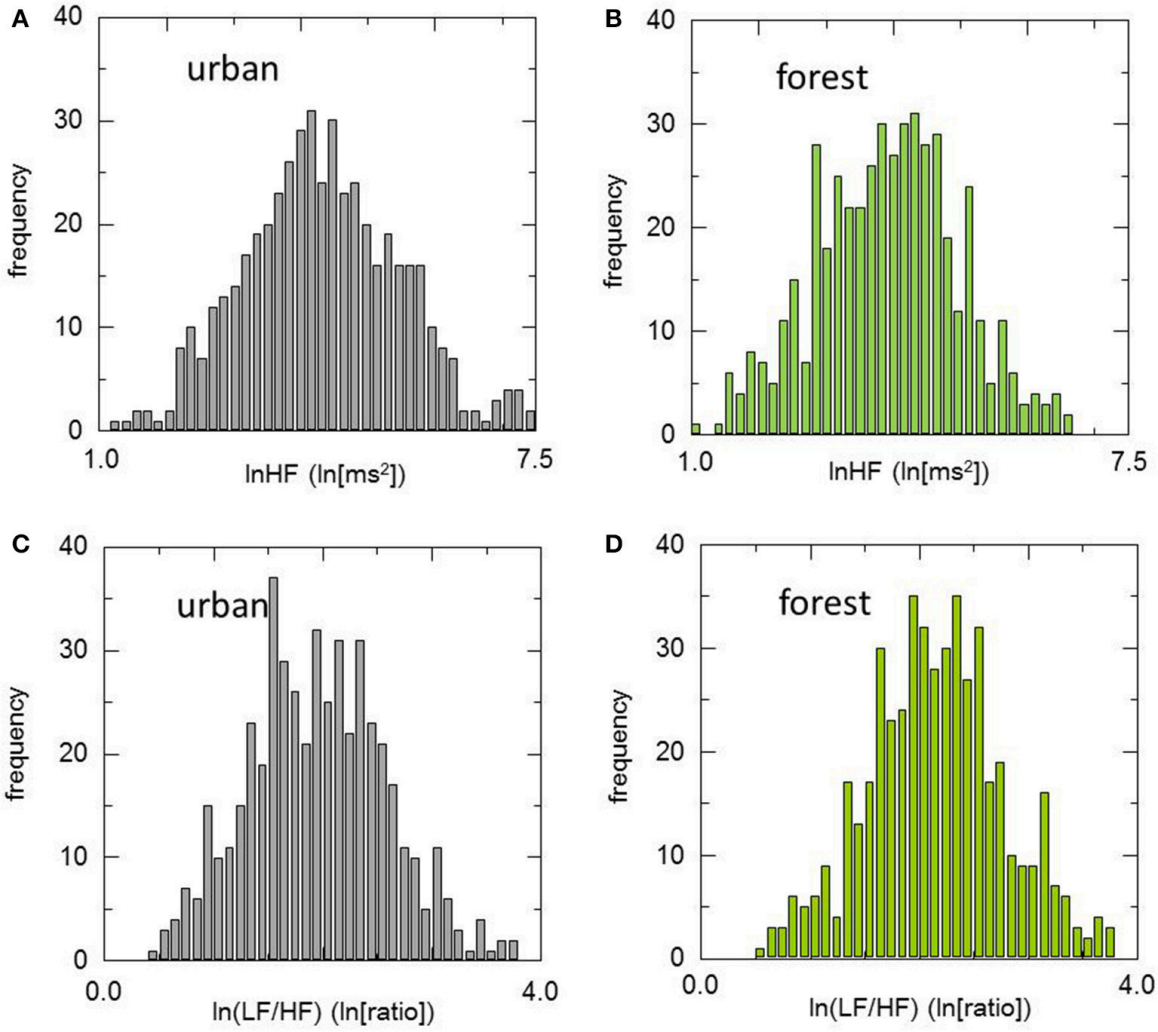
Histograms of heart rate variability during walking in urban and forest environments. Upper panels show the distribution of lnHF in urban **(A)** and forest **(B)** environments, and lower panels show the distribution of ln(LF/HF) in urban **(C)** and forest **(D)** environments. For both lnHF and ln(LF/HF), no significant differences in the shape of the distribution curve were observed between urban and forest environments.

Histograms of HRV indicators during walking in urban and forest environments are shown in Figure 1, and statistics of these indicators are summarized in Table 3. The means of lnHF were 3.93 and 4.33 for the urban and forest environments, respectively. The permutation test revealed that mean lnHF during walking in a forest was significantly larger than during walking in an urban area (*p* < 0.01). The medians of lnHF were 3.96 and 4.27 for the urban and forest environments, respectively, which were also significantly different (*p* < 0.01). Although the difference was not significant (*p* = 0.06), SD was slightly greater in the forest environment than in the urban environment, resulting in CV being almost unchanged (*p* = 0.83). Both Q1 and Q3 were larger in forest walking (*p* < 0.01), and as a result, there was no difference in IQR (*p* = 0.62).

**Table 3:** Distribution characteristics of heart rate variability indices urban and forest environments.

	**lnHF**			**ln(LF/HF)**		
	**Urban**	**Forest**	**Difference(*p*-value)**	**Urban**	**Forest**	**Difference (*p*-value)**
Mean	3.93	4.33	*p* < 0.01	2.16	1.96	*p* < 0.01
Median	3.96	4.27	*p* < 0.01	2.18	1.95	*p* < 0.01
SD	1.06	1.16	*p* = 0.06	0.62	0.63	*p =* 0.55
CV (%)	27.0	26.74	p = 0.83	28.5	32.1	*p* < 0.01
Q1	3.17	3.56	*p* < 0.01	1.74	1.53	*p* < 0.01
Q3	4.65	5.11	*p* < 0.01	2.54	2.39	*p* < 0.01
IQR	1.49	1.55	*p* = 0.62	0.80	0.86	*p =* 0.34
Skewness	−0.03	0.13	*p* = 0.24	0.10	0.18	*p =* 0.47
Kurtosis	−0.30	−0.13	*p* = 0.43	−0.15	−0.24	*p =* 0.64

*SD, standard deviation; CV, coefficient of variation; Q1, quartile 1 (25th percentile); Q3, quartile 3 (75th percentile); IQR, interquartile range; Skewness, a measure of symmetry of distribution; Kurtosis, a measure of whether the distribution curve is peaked (positive) or flat (negative) relative to the normal distribution. Differences between urban and forest environments were tested by a permutation test*.

In regards to In(LF/HF) the means were 2.16 in the urban environment and 1.96 in the forest environment, and significantly larger ln(LF/HF) was observed in the urban environment than in the forest environment (*p* < 0.01). As for the median of Q1 and Q3, the differences between urban and forest areas were statistically significant, but the differences in SD and IQR were not as significant. These results were similar to those of lnHF, although the direction of the change was the opposite. Unlike the results of lnHF, nevertheless, CV of ln(LF/HF) was significantly larger in the forest environment (32.1) than in the urban environment (28.5) (*p* < 0.01).

The mean and median values were very close in both HRV indicators and in both environments. For example, the values were 3.93 (mean) and 3.96 (median) for lnHF in an urban area. This suggested that the distribution curves of this variable were almost symmetric. This symmetrical distribution was also confirmed by higher moment statistics. Skewness and kurtosis were close to zero for both HRV indicators and both environments, suggesting nearly normal distributions.

**Figure 2:**
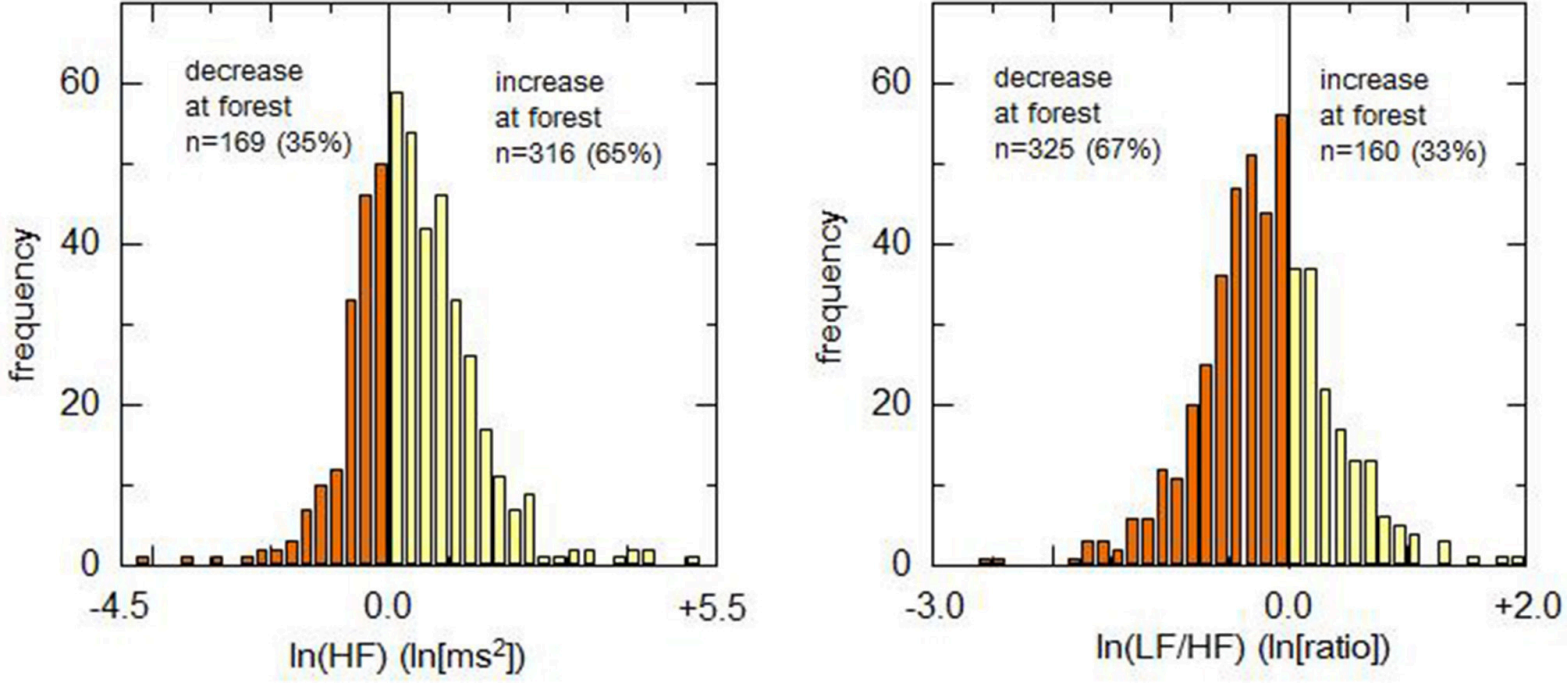
Histograms of difference in heart rate variability indicators between urban and forest environments. Left and right panels demonstrate histograms for the difference in lnHF and ln(LF/HF), respectively. As for the parasympathetic indicator (lnHF), the percentage of positive responders (presenting an increase in forest environment) was ~65%. Regarding the sympathetic indicator [ln(LF/HF)], the percentage of positive responders (presenting a decrease in forest environment) was ~67%.

The differences between urban and forest environments for lnHF and ln(LF/HF) were plotted in a histogram (Figure 2). Positive and negative values in the abscissa represent increases and decreases in the HRV indicator in a forest environment, respectively. Due to an increase in lnHF or a decrease in ln(LF/HF) is considered to represent relaxation, it was defined that these changes are positive responses. Conversely, a decrease in lnHF and an increase in ln(LF/HF) were defined as negative responses. As for lnHF, 316 (65.2%) participants showed positive responses in the forest environment rather than in the urban environment, and the remaining 169 (34.8%) participants exhibited negative responses. The ln(LF/HF), 325 (67.0%) showed decreases in the forest environment and the remaining 160 (33.0%) exhibited negative responses.

The present results of HRV during walking were compared with the previously reported results on HRV during the viewing of landscapes (17). The numbers of participants who indicated positive/negative responses in HRV indicators in a forest are summarized in Table 4. In our previous results (17), 79.2% participants exhibited positive responses (increases in the forest environment) in lnHF during the viewing of landscapes. The proportion of positive responders during viewing was considerably larger than the proportion during walking. A chi-square test revealed significant difference in the proportion of positive responders in lnHF between walking and viewing (*p* < 0.01).

**Table 4:** Number of participants who indicated positive / negative response of HRV indices in forest environment.

	**lnHF**		**ln(LF/HF)**	
	**Positive response**	**Negative response**	**Positive response**	**Negative response**
Walking (*n* = 485)	316 (65.2%)	169 (34.8%)	325 (67.0%)	160 (33.0%)
Viewing^*^ (*n* = 625)	495 (79.2%)	130 (20.8%)	400 (64.0%)	225 (36.0%)
Chi-squared	27.4 (*p* < 0.01)		1.0 (*p* = 0.30)	

* *Results on HRV during viewing urban or forest landscapes were presented in our previous report (17)*.

On the other hand, the proportion of positive responders in ln(LF/HF) during viewing was 64.0%, which was close to the proportion during walking (67.0%) demonstrated in this study. A chi-square test revealed that this difference in ln(LF/HF) was not statistically significant (*p* = 0.30).

## Discussion

### Analysis of distribution characteristics

One of this study's feature is the inclusion of an analysis with special reference to the distribution characteristics of individual variations in the HRV response. Skewness and kurtosis of HRV indices did not change in either lnHF or ln(LF/HF), although significant changes in the mean values were observed between urban and forest environments. In other words, walking in a forest environment shifted the distribution curve higher (lnHF) or lower [ln(LF/HF)] while maintaining its shape. This was similar to the results in HRV during the viewing of urban and forest landscapes in a previously reported study (17).

Not all physiological indicators, however, maintain the shape of their distribution curve in response to natural environments. Salivary cortisol concentration indicated a significant decrease in forest environments compared with that in urban environments, accompanying a more skewed and kurtotic distribution (10). This modification of the distribution curve might be attributed to a floor effect (28, 29). Therefore, an unchanged distribution curve is a specific response in log-transformed HRV indicators.

### Effects of natural environment on HRV

During walking in forest environments, larger lnHF and smaller ln(LF/HF) were observed compared with those upon walking in urban environments. As the lnHF and ln(LF/HF) are indicators of parasympathetic and sympathetic nervous activity, the present results implied that the autonomic relaxation occurred during walking in forest environments. The results are consistent to those in our previous study (17). Therefore, walking in forest environments and viewing forest landscapes demonstrated qualitatively similar effects on autonomic functions.

Controversy, quantitative comparisons between the present and previous results revealed a different tendency in the autonomic response to walking and viewing. During walking in forest environments, 65.2% participants exhibited a positive response in the parasympathetic indicator (lnHF), which was significantly lower than the percentage of positive responders during viewing of forest landscape (79.2%). Contrary, for the sympathetic indicator, the percentage of positive responders during walking (67.0%) was almost identical to that during viewing (64.0%). Therefore, the effect of a forest environment on parasympathetic nervous activity was more apparent during viewing than walking, whereas sympathetic activity exhibited almost the same responses to viewing and walking regarding the percentage of positive responders.

### Positive and negative effects of a natural environment

In 1984, the distinguished biologist Edward O. Wilson proposed the biophilia hypothesis (30). Biophilia is defined as the “innate tendency to focus on life and life-like processes” (31). For millions of years, our ancestors lived in the savannas of Africa. Within this environment, natural features, such as trees or forests, could provide food, water, or shelter, thereby increasing the probability of survival. Thus, biophilia can be regarded as an adaptive characteristic.

Alternatively, it is known that certain people show a strong dislike for natural settings. This tendency is called biophobia (32). Biophobia includes certain specific phobias, such as arachnophobia (irrational fear of spiders) or entomophobia (fear of insects). There is also a term referring to the fear of forests (hylophobia/xylophobia) (33). Biophobia is also an adaptive psychological trait because of inherent dangers in the natural environment (e.g., predators and poisonous organisms). Therefore, the effect of the natural environment on humans is two-sided.

From the perspective of evolutionary psychology, a model for the effects of the natural environment on humans has been proposed, which includes three factors: drive, contentment, and threat (34, 35). Drive includes emotions such as joy, approach, appetite, stimulation, and positiveness. As an endocrine response, it is related to dopamine secretion. In contrast, contentment is concerned with emotions such as calmness, relaxation, and safety and is related to the oxytocin and opiate systems. In terms of autonomic regulation, drive and contentment are associated with sympathetic and parasympathetic activities, respectively (35). A relaxation in autonomic nervous activity [increase in lnHF and decrease in ln(LF/HF)] was observed in the forest environment during both walking and viewing; therefore, it can be considered that exposure to a forest environment mainly confers contentment rather than drive. Furthermore, a comparison between present and our previous results suggested that viewing a forest landscape could provide more contentment than walking in a forest environment.

A major limitation of this study is that it included only Japanese young male subjects. The tendency for biophilia/biophobia may be affected by difference in age, gender, and ethnicity of participants. Effects of demographic and geographic factors on physiological responses to natural environments should be investigated in a future study.

## Conclusion

The autonomic relaxation (increases in parasympathetic indicator and/or decreases in sympathetic indicator) in forest environments has been demonstrated by HRV analysis in previous studies. This result was also confirmed in this study. However, a comparison between the present and our previous study (17) suggested that the response of HRV differ between viewing and walking.

The effect of forest environments consists of several factors, including negative emotions. It is reasonable that a certain percentage of a population exhibits a negative response to forest environments. Therefore, population-based analysis is required in which the existence of negative responders is taken into consideration.

## Author contributions

HK contributed to statistical analysis, interpretation of the results, and manuscript preparation. CS and HI were involved with data acquisition and initial analysis of the results. B-JP, JL, and TK participated in data acquisition and study design. YM had an important role in the research, particularly in experimental design, data acquisition, and manuscript preparation. All authors contributed to the preparation of the manuscript and are responsible for the final editing and approval.

### Conflict of interest statement

The authors declare that the research was conducted in the absence of any commercial or financial relationships that could be construed as a potential conflict of interest.
